# Home Safety Evaluation Model for Older Adults With Recurrent Falls

**DOI:** 10.1093/geroni/igaa057.850

**Published:** 2020-12-16

**Authors:** Willy Marcos Valencia, Kimberly Cabrera, Vincent Hsu

**Affiliations:** 1 Miami VA Healthcare System, Miami, Florida, United States; 2 Miami VAMC, Miami, Florida, United States; 3 University of Miami Miller School of Medicine, Miami, Florida, United States

## Abstract

Recurrent falls are a major threat in older adults. Home environment can be a hazard, but it is potentially modifiable/reversible. In Miami VA, occupational therapists conduct home safety evaluations (HSE) to ascertain the need for modifications to reduce falls risk. We reviewed the cohort of high-risk, recurrent falls patients evaluated at our Falls Prevention Clinic (FPC) between August 2017 to November 2019, to evaluate the impact of HSE. We identified 48 Veterans, age 76.5±6.9 years, of whom 15 (31.3%) reported 1-2 falls/year, 18 (37.5%) reported 3-4 falls/year, and 15 (31.3%) reported ≥5 falls/year. Twenty-eight (58.3%) were offered a HSE. Within these subjects, 74.2% reported falling at least once within their home, 43.8% had fear of falling, 5 (17.9%) had a history of substance or alcohol abuse. We observed that 29 (60.4%) would benefit from the addition of grab bars and 26 (54.2%) could benefit from toilet adjustments. Twelve (25.0%) were recommended to install bed rails. Only 15 (31.3%) Veterans agreed to all recommendations, 25 (52.1%) declined due to preference, and 8 (16.7%) declined for other reasons. Only 8 (16.7%) of these Veterans lived alone. Another factor is that 11 (22.9%) Veterans were renting and 32 (66.7%) owned their homes. Addressing and improving environmental hazards may ameliorate the risk for recurrent falls. Our next steps are to evaluate the extent of home modifications, and the long-term changes in falls/year. Further research needs to determine the long-term efficacy and cost-effectiveness of HSE, and how it can be more accessible to the community.

